# Large-scale molecular epidemiological survey of *Giardia* and *Cryptosporidium* in Victoria, Australia (2020–2024), reveals novel subtypes and outbreak-associated lineages

**DOI:** 10.1128/jcm.01558-25

**Published:** 2026-03-27

**Authors:** Marielle Babineau, Anson V. Koehler, Michelle L. Sait, Karolina Mercoulia, Sally Dougall, Jane McAllister, Evelyn Wong, Norelle L. Sherry, Robin B. Gasser, Benjamin P. Howden

**Affiliations:** 1 Department of Microbiology and Immunology, University of Melbourne at the Peter Doherty Institute for Infection and Immunity, The University of Melbourne 2281, Melbourne, Victoria, Australia; 2 Department of Veterinary Biosciences, Melbourne Veterinary School, The University of Melbourne 2281, Parkville, Victoria, Australia; 3 Microbiological Diagnostic Unit Public Health Laboratory, Department of Microbiology & Immunology, University of Melbourne at the Peter Doherty Institute for Infection and Immunity, Melbourne, Victoria, Australia; 4 Department of Health 1457 , Melbourne, Victoria, Australia; 5 Centre for Pathogen Genomics, The University of Melbourne 2281, Melbourne, Victoria, Australia; Mayo Clinic Minnesota, Rochester, Minnesota, USA

**Keywords:** *Cryptosporidium*, *Giardia*, molecular epidemiology, notifiable disease, public health surveillance, zoonosis, gastrointestinal infection, outbreak detection, Australia

## Abstract

*Cryptosporidium* and *Giardia* are major causes of gastrointestinal illness globally. In Australia, cryptosporidiosis is nationally notifiable, yet molecular characterization is not routinely undertaken, limiting outbreak detection, source attribution, and zoonotic risk assessment. This limitation became apparent in 2024, when Australia experienced a 273% increase in cryptosporidiosis notifications, the third-highest rise globally. We conducted a molecular epidemiological investigation of *Cryptosporidium* and *Giardia* in fecal samples from humans with gastroenteritis in Victoria, Australia, between 2020 and 2024. Positive samples were sequenced using *SSU* and *gp60* for *Cryptosporidium* and *tpi* for *Giardia*, and parasite load was estimated. Of the 2,330 samples tested, 225 were positive for *Cryptosporidium* and nine for *Giardia*. One *Giardia* sample was identified as sub-assemblage AI, two as AII, and six as assemblage B. Seven *Cryptosporidium* species and 24 subtypes were identified, including eight novel subtypes. *Cryptosporidium hominis* predominated (85%), followed by *C. parvum* and *C. meleagridis*. Six *C. hominis* subtypes were detected, three associated with 11 recreational water-linked outbreaks in 2024. Subtypes IaA12R3 and IeA11G3T3 were most frequent, in line with global trends; concordance analysis indicated that at least 52 additional cases were likely linked to outbreaks. Several *C. parvum* subtypes were identified, including two linked to childcare and camp outbreaks. Novel human-infective subtypes of *C. occultus*, *C. fayeri*, and *C. meleagridis* were also detected. These findings reveal extensive *Cryptosporidium* diversity in Victoria and highlight the value of molecular surveillance for resolving transmission pathways and informing public health responses.

**IMPORTANCE:**

*Cryptosporidium* is a nationally notifiable pathogen in Australia, yet routine subtyping is not performed, limiting the detection of both outbreak-related and sporadic transmission and constraining source attribution. This study represents the first large-scale molecular surveillance of human *Cryptosporidium* and *Giardia* infections in southern Australia, including the first molecular epidemiological exploration of the marked increase in cryptosporidiosis observed in 2024. By integrating species, subtype, or assemblage information with epidemiological metadata, we demonstrate substantial *Cryptosporidium* lineage diversity in symptomatic patients, including multiple novel subtypes and zoonotic transmission. These findings demonstrate the critical value of molecular tools for uncovering hidden transmission, resolving pathways contributing to both sporadic disease and outbreak, and informing targeted public health responses. The findings provide a strong foundation for incorporating routine genotyping into national surveillance systems for parasitic eukaryotic enteropathogens, and the accompanying international comparison of case trends suggests broader transmission dynamics of significant public health relevance.

## INTRODUCTION

*Cryptosporidium* and *Giardia* are among the commonest protistan pathogens causing gastrointestinal illnesses globally, associated with substantial disease burden in both developed and developing countries (1, 2). Cryptosporidiosis is a nationally notifiable disease in Australia, whereas giardiasis is only notifiable in some states and territories and remains underreported and often unquantified. Recent surveillance studies have documented marked increases in cryptosporidiosis notifications in 2023 and 2024 in Germany, Norway, Belgium, Spain, France, and England (3–8). In Australia, an increase in cases linked to swimming pools has been observed, leading to multiple health warnings from public health authorities (9–12). However, the reason for the increase in cryptosporidiosis cases in Australia has not yet been comprehensively investigated. A comparison of cases between Australia and other countries during this period has also not been explored. Such a comparison might allow the magnitude of the outbreaks locally and abroad to be quantified, which could identify large-scale patterns of transmission, subtype-specific disease dynamics, or common risk factors.

Cryptosporidiosis and giardiasis are primarily transmitted via the faecal–oral route, often through contaminated recreational or drinking water (13, 14). In Australia, infections occur predominantly in children and display marked seasonal peaks during warmer months (15–17). Most reported outbreaks have occurred in urban areas and are linked to recreational water exposure (18–30), although sporadic cases and occasional outbreaks linked to animals (31, 32), drinking water (33–35), unpasteurized milk (36, 37), and overseas travel (38, 39) have also been documented.

Routine clinical diagnosis for *Cryptosporidium* and *Giardia* in Australia relies on qualitative detection in fecal samples using PCR or microscopy, which are unable to differentiate between species and subtypes. Currently, there is no systematic referral of test-positive samples to reference laboratories for further genetic characterization, limiting the ability to identify potential outbreaks and to guide targeted public health interventions. This has impeded efforts to resolve transmission pathways, identify outbreak sources, and characterize the diversity of human-infective species, genotypes, and subtypes. A wide range of *Cryptosporidium* and *Giardia* species and subtypes are known to infect humans, each differing in host range, ecological niche, and potential for zoonotic or waterborne transmission (13, 14, 40). To date, 46 *Cryptosporidium* species have been described, with 19 species and more than 60 subtypes reported in humans globally (41, 42). In Victoria, only four species and 10 subtypes have previously been recorded from humans (43, 44). There is no comprehensive study of *Cryptosporidium* subtypes in clinical cases in southern Australia. The identity of *Cryptosporidium* species and subtypes infecting humans remains largely unknown.

Of the eight recognized *Giardia* species, *G. duodenalis* is the principal agent of human infection, with sub-assemblages AI and AII (assemblage A) and assemblage B being most common in humans (40, 45, 46). While these assemblages have been reported in human cases in other states of Australia (47–55), no published data exist on human *Giardia* assemblages in southern Australia, limiting our ability to understand transmission dynamics, identify potential zoonotic sources, and assess local public health risk.

In contrast to the limited data for humans, *Cryptosporidium* and *Giardia* species and subtypes have been extensively characterized in non-human hosts across Australia, including wildlife, livestock, and companion animals (56–61). In Victoria alone, 24 *Cryptosporidium* species and 31 subtypes have been reported from 16 animal hosts (43, 44, 62–68). *Giardia* assemblages have similarly been recorded in a range of animals in Victoria and nationally (52, 54, 63, 64, 69). This disparity in surveillance limits the assessment of zoonotic risk and obscures the true sources of infection in human populations.

Certain subtypes have been associated with specific transmission routes and risk factors. For example, *C. cuniculus* and *C. fayeri* subtypes have been linked to wildlife contact; *C. hominis* subtypes IbA10G2, IbA12G3, IgA17, IfA12G1R5, and IdA15G1 are frequently associated with recreational water outbreaks (5, 30, 31, 70–73); and *C. parvum* subtypes are often associated with livestock exposure (71, 74). Accurate species and subtype identification is essential for outbreak resolution, source attribution, and the assessment of resistance to antimicrobial drugs. The most widely applied molecular targets for species identification are the small subunit rRNA (*SSU*) and subtyping via the 60 kDa glycoprotein (*gp60*) loci for *Cryptosporidium* and the triosephosphate isomerase (*tpi*) locus for *Giardia* (13, 14, 40). However, these methods have not yet been widely applied to understand outbreaks and transmission or to guide public health policies and interventions.

In this study, we retrospectively analyzed a large panel of fecal samples from cases of gastrointestinal illness in Victoria using molecular and phylogenetic methods to detect and genetically characterize *Cryptosporidium* and *Giardia*. We then classified all current and retrospectively identified positive samples at the species, genotype, and subtype or assemblage levels and quantified oocyst or cyst excretion rates to estimate parasite load. Finally, we integrated molecular findings with epidemiological metadata to delineate transmission patterns, assess zoonotic contributions, distinguish sporadic from outbreak-associated cases, and identify potential sources and clusters of infection during the 2020–2024 period.

## MATERIALS AND METHODS

### Sample set

A total of 2,330 human fecal samples were available to test for *Cryptosporidium* and *Giardia* and genetically characterize these protists (Table 1). A total of 2,329 samples were received at the Microbiological Diagnostic Unit Public Health Laboratory (MDU PHL), Melbourne, between 2020 and 2024 and originated from residents of Victoria (population 6.9 million in 2024 [75]), Australia. One additional *Giardia*-positive sample from 2018 was also included. Samples were categorized as (i) *Cryptosporidium*-positive (*n* = 219), (ii) *Giardia*-positive (*n* = 9), and (iii) untested samples from symptomatic patients (*n* = 2,102).

**TABLE 1:** Number of human clinical fecal samples processed by year, sample type, and detection rate of *Cryptosporidium* and *Giardia*^*a*^

Year	Sample type and results from diagnostic testing	No. of samples screened (this study)	No. (%) of *Cryptosporidium* positives	No. (%) of *Giardia* positives
2018	Detected – *Giardia*	1	0	1 (100%)
2020	Detected – *Cryptosporidium*	0	0	0
	Detected – *Giardia*	3	0	0 (0%)
	Untested	234	3 (1.3%)	1 (0.4%)
2021	Detected – *Cryptosporidium*	1	1 (100%)	0
	Detected *– Giardia*	3	0	0 (0%)
	Untested	544	1 (0.2%)	2 (0.3%)
2022	Detected – *Cryptosporidium*	1	0	0
	Detected *– Giardia*	1	0	1 (100%)
	Untested	439	0	1 (0.2%)
2023	Detected – *Cryptosporidium*	0	0	0
	Detected *– Giardia*	1	0	1 (100%)
	Untested	658	0	0
2024	Detected – *Cryptosporidium*	217	214 (98.6%)	1 (0.5%)
	Detected *– Giardia*	0	0	0
	Untested	227	6 (2.6%)	1 (0.4%)
Total		2,330	225 (9.6%)	9 (0.4%)

^*a*^ Detected – *Cryptosporidium*: samples in which *Cryptosporidium* was previously detected by PCR or microscopy. Detected – *Giardia*: samples in which *Giardia* was previously detected by PCR or microscopy. Untested: samples from patients with gastroenteric symptoms previously not tested for *Cryptosporidium* or *Giardia*.

Notified case specimens are not currently routinely referred for further molecular analysis, but for the purposes of this study, *Cryptosporidium*-positive fresh human fecal samples (identified by various multiplex PCR and/or fixed stool microscopy) in 2024 were submitted to MDU PHL from multiple diagnostic laboratories within 2–4 weeks after detection. Prior to 2024, the *Cryptosporidium* and *Giardia* positive samples held at MDU PHL were initially identified using the PCR-based BD MAX Enteric Parasite Panel (Becton Dickinson, USA). Untested samples are routinely submitted from individuals with symptoms of gastroenteritis, with the majority of these associated with notified gastroenteritis outbreaks, possibly from a foodborne or waterborne source, but not previously tested for *Cryptosporidium* or *Giardia*. All samples were received and stored unpreserved at –20°C (2024 samples) or –70°C (<2024) before processing.

### Isolation of genomic DNA

Fecal samples were thawed at 4°C and homogenized using a FastPrep−96 (MP Biomedicals) at 1,400 rpm for 10 min. Total genomic DNA was extracted using the QIAamp PowerFecal Pro DNA Kit on the QIAsymphony SP platform (Qiagen, Germany), following the manufacturer’s protocol. Between 0.1 and 0.4  g of human fecal material (mean: 0.25  g) was used per extraction. To monitor PCR inhibition and DNA recovery, qPCR Extraction Control Red (Meridian Bioscience, USA) was spiked into 1,571 samples across all years. Eluted DNA (85  µL) was stored at –20°C. No-template extraction controls were included in each batch (48–96 samples).

### PCR and Sanger sequencing

Nested PCR was used to amplify *Cryptosporidium* small subunit rRNA (*SSU*) and 60 kDa glycoprotein gene (*gp60*) loci, and *Giardia* triosephosphate isomerase (*tpi*) loci, using GoTaq G2 Hot Start Taq polymerase (Promega, USA) and established protocols (64). *SSU* primers were sourced from previous publications (76–78) and yielded ~800  bp amplicons. Amplification of *gp60* from *C. meleagridis* or *C. occultus* followed established methods (79, 80). Amplicons were treated with ExoSAP-IT Express (Applied Biosystems, USA) and sequenced bidirectionally using BigDye Terminator v3.1 chemistry (Applied Biosystems). Chromatograms were inspected and trimmed manually using Geneious Prime 2025 v2.1 (81).

### Phylogenetic analysis

Sequences were aligned using MUSCLE (82) in Geneious and manually refined. Sequence identities were assessed via BLASTn using GenBank. Subtypes of *gp60* were assigned according to established nomenclature (80, 83). Substitution models were selected using MEGA v11.0.13 (84) based on the lowest AIC score: GTR+G+I for *SSU* and *gp60*; K2+G+I for *tpi*. Bayesian inference was performed using MrBayes v3.2.7 (85) with 10–50 million generations (depending on locus), four chains, and sampling every 1,000 generations. The first 25% of trees were discarded as burn-in. Convergence was confirmed by split frequency standard deviation (<0.01) and a scale reduction factor of 1.0. Outgroups included *C. muris*, *C. fayeri*, and *G. muris*. Phylogenetic trees were visualized and annotated using iTOL (86).

### Quantification of oocysts and cysts by multiplex qPCR

A validated multiplex qPCR assay (see the supplemental methods) was used to estimate *Cryptosporidium* oocyst and *Giardia* cyst numbers (per gram of feces) in PCR test-positive samples. The assay targeted *SSU* (*Cryptosporidium*) and the glutathione dehydrogenase (*gdh*) gene (*Giardia*) and was calibrated using standard curves from serially diluted DNA derived from *C. parvum* subtype IIaA17G2R1 from Bunchgrass Farms and *G. duodenalis* wild-type strain WB1B. Samples were screened in triplicate, and samples with Ct values of <37 were retained. The mean genome copy number per gram was calculated. For *Cryptosporidium*, the qPCR-based estimates were cross-validated with oocyst counts from immunomagnetic separation followed by direct enumeration in a hemocytometer. Assay development and validation protocols are described in the supplemental methods.

### Epidemiological data analysis

Epidemiological data were obtained from the Victorian Department of Health for all notified cases of cryptosporidiosis between 2019 and 2024. Data included patient age, sex, hospitalization status, notification and sample collection dates, outbreak linkage, outbreak setting, and outbreak ID code. Age (in years) was stratified into 10-year brackets. Cases were categorized as hospitalized if they were admitted to a hospital or presented to the hospital emergency department. The sample postcodes were matched to local government areas (LGAs) using the Australian Statistical Geography Standard (Australian Bureau of Statistics, July 2011). If a postcode spanned multiple LGAs, the first listed alphabetically was selected. Spatial, temporal, and subtype data were used to identify additional cases potentially linked to outbreaks. If a case could be potentially linked to multiple outbreaks, the earliest outbreak was selected. Travel history data and the immune status of the notified cases were not obtained. Associations between *Cryptosporidium* oocyst load, species, and subtypes, and patient age, sex, hospitalization status, and outbreak setting were analyzed using ANOVA.

### Cryptosporidiosis cases nationally and internationally

The annual summary of cryptosporidiosis cases from 2019 to 2024 was obtained from a total of 32 countries from North America (the USA and Canada), Oceania (all jurisdictions within Australia and New Zealand), and 28 countries in Europe, including the European Union as a whole (see the supplemental methods). Regions with fewer than 10 cases in 2023 or 2024 were excluded. Regions with no data for two or more years during the period were excluded. To the best of our knowledge, the regions identified above are the only ones worldwide where cryptosporidiosis is a reportable disease and that have a surveillance system with publicly available data. Regions were classified into three categories based on the increase in cases observed in 2023 or 2024 compared to the previous year: sharp (>50% increase in cases), moderate (15–50% increase), and none (<15%). The percentage of increase for 2023 or 2024 was calculated as: ((latest year case number − previous year case number)/previous year case number) × 100. Comparison of the case numbers for 2023/2024 against the mean of the four previous years was misleading due to the global decrease in cases during the COVID-19 pandemic. The comparison of 2023 in the Northern Hemisphere with 2024 in the Southern Hemisphere reflects the shift in the high-risk summer season across those regions.

## RESULTS

### Detection of parasites in human fecal samples

Of 2,330 human fecal samples tested, 225 were test-positive for *Cryptosporidium* and nine for *Giardia* by PCR-based sequencing (Table 1). One sample from 2024 showed a mixed infection of *Cryptosporidium* and *Giardia*. The detection rate for previously confirmed *Cryptosporidium*-positive samples was 98%. For untested samples, detection ranged from 0% in 2022–2023 to 2.6% in 2024 (Table 1). For *Giardia*, the detection rate was 33% for previously confirmed positives and 0.3% for untested samples.

### *Cryptosporidium hominis* is the most common species identified among six others

Seven *Cryptosporidium* species were identified using *SSU*: *C. hominis* (*n* = 191), *C. parvum* (*n* = 25), *C. meleagridis* (*n* = 3), *C*. sp. mink genotype (*n* = 3), *C. fayeri* (*n* = 1), *C. occultus* (*n* = 1), and *C*. sp. OTUi (*n* = 1) (Fig. S1). All *C. hominis SSU* sequences were identical. All *C. parvum* sequences were identical to the IOWA strain (OR421305). One *C. meleagridis* sequence had a single T>A substitution; all three were otherwise identical and matched the UKMEL3 reference (87). The *C*. sp. mink genotype sequences (*n* = 3, from 2020) were identical and matched two shorter Australian human-derived sequences (88, 89). These human sequences clustered in a polytomy distinct from animal-derived sequences, which grouped with *C. sciurinum*. The conserved nature of the *SSU* locus limited interspecies resolution.

The *Cryptosporidium* sp. OTUi sequence (2024) matched sequences from a returned traveler and a bat from the Philippines (38, 90) and was 98.9% similar to *C. wrairi*. The *C. fayeri* sequence matched one from a kangaroo in Victoria (64). The *C. occultus* sequence (2021) was identical to that of a deer in Victoria and contained a species-specific motif distinguishing it from *C. suis* (91).

### High intraspecific *gp60* subtype diversity and novelty

Ten *gp60* subtypes were detected among *C. hominis* samples: IaA12R3 (*n* = 54), IaA14R3 (*n* = 3), IaA16R3 (*n* = 1), IaA16R4 (*n* = 2), IaA24R1 (*n* = 1), IbA8G4 (*n* = 1), IdA15G1 (*n* = 17), IeA11G3T3 (*n* = 96), IfA12G1R5 (*n* = 13), and IfA13G1R4 (*n* = 1) for a total of 189 sequences (Fig. S2). Two samples were not amplified. All sequences within each subtype were identical. Several subtypes matched previously reported sequences in Australian or international data sets (e.g., IaA12R3, IdA15G1, IeA11G3T3, IfA12G1R5), while IbA8G4 showed only 90–91% identity to other Australian Ib subtypes. Subtype clades formed strongly supported monophyletic groups within subfamilies but lacked resolution at deeper nodes.

In the 25 samples containing *C. parvum*, seven *gp60* subtypes were detected: IIaA16G1R1 (*n* = 1), IIaA16G3R1 (*n* = 1), IIaA17G2R1 (*n* = 3), IIaA18G3R1 (*n* = 9), IIaA19G3R1 (*n* = 4), IIaA19G4R1 (*n* = 3), and IIaA20G3R1 (*n* = 2) (Fig. S3). Two samples were not amplified. All sequences within each subtype were identical. Most matched cattle- or wildlife-derived Australian sequences (32, 62, 92), while IIaA16G1R1 matched cattle sequences from overseas (93).

**Fig 1:**
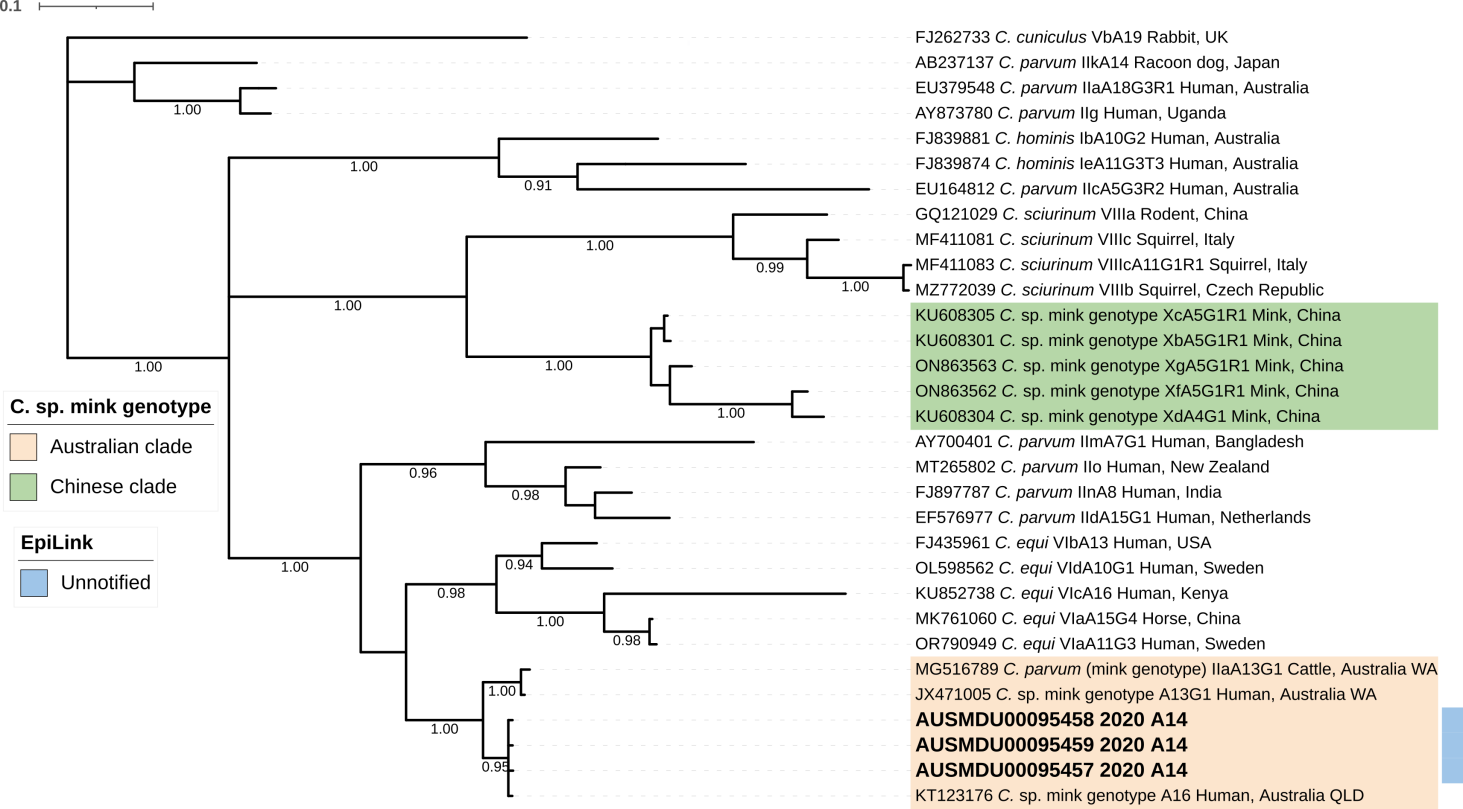
Phylogenetic tree reconstructed from the Bayesian topology using GTR + G + I model based on 60 kD glycoprotein gene (*gp60*) with 10 M generations for *C*. sp. mink genotype sequences. Sequences generated in this study are shown in bold. Posterior probabilities equal to or above 0.90 are shown below the branch. *Cryptosporidium cuniculus* is used as outgroup.

Three *gp60* sequences from *C*. sp. mink genotype (subtype A14) were identical to each other and to prior human-derived sequences. One previously published sequence from cattle clustered within the same clade. These sequences were distinct from non-Australian mink sequences, supporting a regional divergence. All non-Australian *C*. sp. mink genotype *gp60* sequences available originate from China. The sequences were more closely related to *C. equi* (Fig. 1).

Three *C. meleagridis* subtypes were identified: IIIjA4T1, IIIA19G1, and IIIgA31G4 (Fig. S4). IIIjA4T1 was 88% similar to a wallaby-derived sequence (94). The IIIA19G1 sequence was not assignable to a known subfamily and contained a unique 33 bp insertion. IIIgA31G4 was 98% similar to a sequence from a human in Sweden (79).

The *C. fayeri* subtype IVfA10G1T1 was 98% similar to sequences from kangaroos (92, 95). The *C*. sp. OTUi *gp60* sequence (subtype A12G1) was 96% similar to a 2014 human-derived sequence (A15G1) and showed a synonymous SNP and a three-repeat deletion. The *C. occultus* subtype was identified as XXIVa and was most similar (94%) to a sequence from a Swedish rat (GenBank accession number PV067140).

### Three *Giardia tpi* assemblages identified

Nine sequences representing *Giardia duodenalis* were assigned to sub-assemblages AI (*n* = 1), AII (*n* = 2), and assemblage B (*n* = 6) (Fig. S5). Six were from previously untested symptomatic patients. The AI sequence matched the reference genome strain WB C6 and sequences from a rabbit and a kangaroo in Victoria (64). The AII sequences were identical to a previously reported Australian human-derived sequence (89). Four assemblage B sequences matched samples from Spain and the US wastewater (96) and showed high similarity (99.1%) to international human-derived sequences.

### Oocyst and cyst load are independent of subtypes

The 225 *Cryptosporidium* test-positive samples were subjected to qPCR to estimate parasite load; 194 were successful (Table 2). Of the excluded 31 samples, 22 yielded no Ct value, and nine had Ct > 37. Oocyst load did not differ significantly by species (F = 0.565, *P* = 0.758) or subtype (F = 1.247, *P* = 0.212). Only one *Giardia*-positive sample (from 2018) yielded quantifiable cysts; the remainder failed amplification (*n* = 3) or exceeded the Ct threshold. Of the five *Giardia* samples which included the qPCR spiked control (collected in 2020, one in 2021, 2024, and 2022), two showed amplification inhibition (collected in 2020 and 2022). Amplification inhibition was not observed from two of the four *Cryptosporidium* samples, which tested negative; the remaining two samples were depleted and could not be assessed for inhibition. Cyst load in the *Giardia* sample was low relative to *Cryptosporidium*. qPCR-based estimates of oocyst load differed by 1–2% from hemocytometer counts following purification via immunomagnetic separation.

**TABLE 2:** *Cryptosporidium* subtypes and *Giardia* sub-assemblages found in this study with respective oocysts and cysts, referred to as oo/cysts, load quantification based on multiplex qPCR^*a*^

Genus	Species	Subtype	No. of samples quantified/ total no. of samples (%)	Mean oo/cysts per gram (standard deviation)
*Cryptosporidium*	*hominis*		165/191 (86%)	1,137,111 (3,097,661)
		IaA12R3	45/54 (83%)	798,686 (2,021,049)
		IaA14R2	3/3 (100%)	1,423,028 (2,455,139)
		IaA16R4	2/2 (100%)	1,104,387 (1,100,996)
		IaA16R3	0/1	
		IaA24R1	0/1	
		IbA8G4	1/1 (100%)	13,353,719 (NA)^*b*^
		IdA15G1	16/17 (94%)	445,441 (1,341,718)
		IeA11G3T3	83/96 (86%)	1,286,463 (3,665,980)
		IfA12G1R5	12/13 (92%)	1,464,549 (2,747,551)
		IfA13G1R4	1/1 (100%)	235 (NA)
	*parvum*		21/25 (84%)	159,452 (400,238)
		IIaA16G1R1	1/1 (100%)	6,596 (NA)
		IIaA16G3R1	0/1	
		IIaA17G2R1	3/3 (100%)	618,394 (1,062,417)
		IIaA18G3R1	9/9 (100%)	96,656 (128,619)
		IIaA19G3R1	3/4 (75%)	111,636 (163,987)
		IIaA19G4R1	2/3 (66%)	91,071 (23,256)
		IIaA20G3R1	2/2 (100%)	49,455 (58,151)
	*meleagridis*		2/3 (66%)	14,743 (11,229)
		IIIgA31G4R1	1/1 (100%)	6,803 (NA)
		IIIjA4T1	0/1	
		III A19G1R1	1/1 (100%)	22,683 (NA)
	sp. mink genotype	A14	3/3 (100%)	20,292 (23,076)
	*fayeri*	IVfA10G1T1	1/1 (100%)	3,975 (NA)
	*occultus*	XXIVa	1/1 (100%)	72 (NA)
	sp. OTUi-like	A12G1	1/1 (100%)	184,663 (NA)
*Giardia*	*duodenalis*	AII	1/2 (50%)	375 (NA)
		AI	0/1	
		B	0/6	

^*a*^ Two *C. hominis* samples and one *C. parvum* sample could not be assigned a subtype but were successfully quantified.

^*b*^ NA, not available.

### Subtyping differentiates between swimming pool outbreaks and other outbreak settings

Of 225 *Cryptosporidium*-positive cases, 173 were notified, and 52 were previously not notified to public health authorities. Six of the notified cases were notified for a foodborne or waterborne illness other than cryptosporidiosis, as they were part of the untested category of samples from 2024; no epidemiological data were available for these six cases. Two cases were positive for rotavirus, one was positive for norovirus, and no bacterial or viral pathogens were detected in the other four cases. Four of the non-notified cases, the three *C*. sp. mink genotype and *C. occultus* cases, were newly detected in this study.

**Fig 2:**
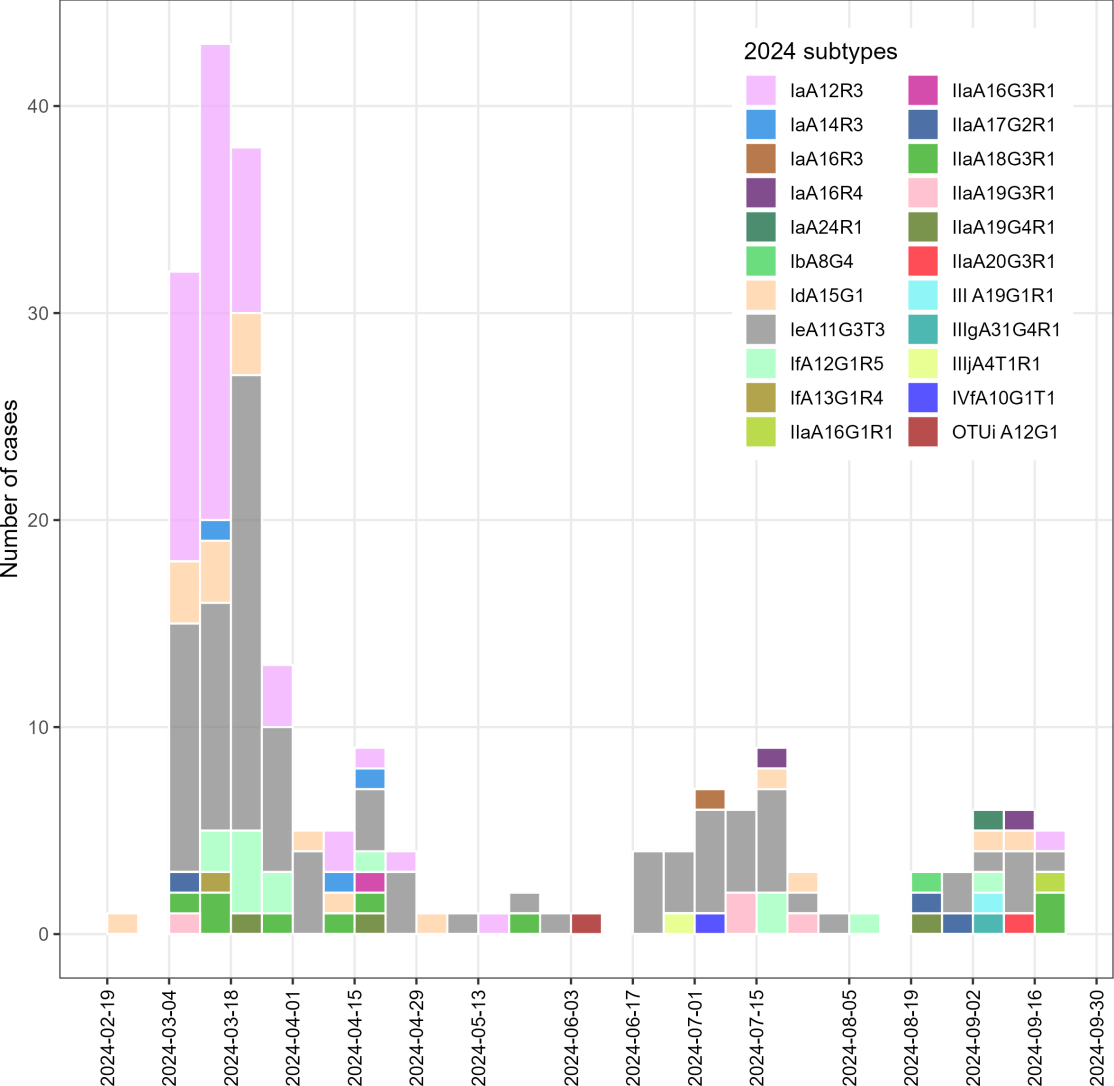
Epidemiological curve of 216 subtyped cases of cryptosporidiosis in 2024 in Victoria.

The 216 subtyped samples from 2024 represented 6.6% of all notified cryptosporidiosis cases in Victoria that year (notified between 19 February and 18 September; Fig. 2). Children <10 years accounted for 95 cases, followed by adults aged 31–40 (*n* = 51). Females comprised 65% (*n* = 143) of subtyped cases. Subtype identity was not correlated to patient age (F = 0.57, df = 3, *P* = 0.7).

Among the 167 cryptosporidiosis-notified cases with subtype data, 140 (84%) were classified as sporadic, and 26 (16%) were epidemiologically linked to 13 outbreaks out of a total of 39 outbreaks ongoing or declared in 2024 in Victoria (11 outbreaks in swimming pools, one outbreak in a childcare, one outbreak in a camp; Table 3; Fig. S2 and S3). Oocyst load was higher in children <10 years (F = 4.8, df = 201, *P* = 0.03). Oocyst excretion rate was similar between sporadic and outbreak-linked cases, including swimming pool outbreak cases (F = 2.9, df = 2, *P* = 0.06). No significant difference in oocyst load was observed between sexes (F = 2.1, df = 1, *P* = 0.15) or hospitalization status (F = 0.2, df = 4, *P* = 0.91). Of the 167 cryptosporidiosis-notified and subtyped cases, 16 cases (8 females, 8 males) were hospitalized, 15 of which were infected with *C. hominis*—IeA11G3T3 (*n* = 10), IaA12R3 (*n* = 2), IaA14R3 (*n* = 1), IfA12G1R5 (*n* = 1), and unknown subtype (*n* = 1)—and the remaining case was infected with *C. parvum* (unknown subtype). Only three hospitalized cases were below the age of 10 years old, and the majority of subtyped hospitalized cases (*n* = 12) were between the ages of 21 and 40 years.

**TABLE 3:** Cryptosporidiosis cases subtyped and epidemiologically linked to outbreaks in Victoria from 2019 to 2024^*a*^

Year	Outbreak ID	Setting	Outbreak length (days)	Total no. of cases (cases subtyped)	Subtype identified	Possible additional cases
2024	o4	Pool	119	38 (3)	IeA11G3T3 (*n* = 1), IaA12R3 (*n* = 2)	15, IeA11G3T3 (*n* = 12), IaA12R3 (*n* = 3)
	o6	Pool	59	27 (1)	IaA12R3	3
	o12	Pool	94	21 (7)	IaA12R3 (*n* = 6), IdA15G1 (*n* = 1)	25, IaA12R3 (*n* = 22), IdA15G1 (*n* = 3)
	o13	Pool	34	25 (4)	IaA12R3	1
	o17	Pool	71	7 (1)	IeA11G3T3	0
	o18	Pool	35	12 (1)	IeA11G3T3	5
	o20	Pool	40	7 (2)	IeA11G3T3	2
	o21	Pool	43	4 (1)	IaA12R3	0
	o22	Pool	47	12 (2)	IeA11G3T3	0
	o23	Pool	6	4 (1)	IeA11G3T3	1
	o34	Childcare	18	4 (1)	IIaA19G3R1	0
	o37	Pool	21	4 (1)	IeA11G3T3	0
2021	o1	Camp	2	6 (1)	IIaA20G3R1	0

^*a*^ Possible additional cases are based on cases with the same subtype, notified during the same outbreak period from residents of the same, or directly adjacent, local government areas.

**Fig 3:**
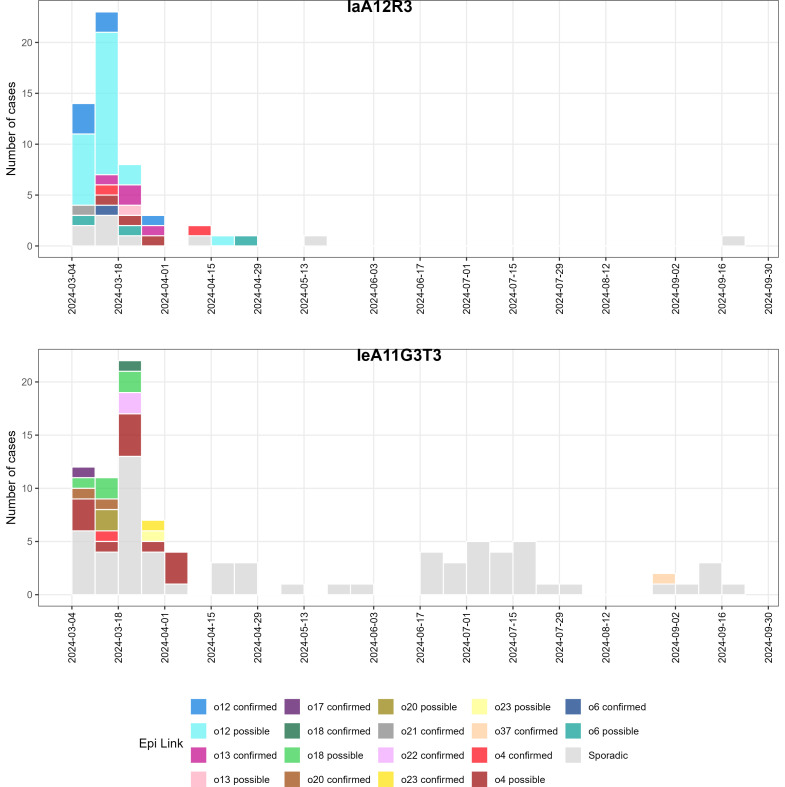
Epidemiological curve of cryptosporidiosis subtype IaA12R3 and IeA11G3T3 cases identified in 2024 in Victoria, classified by epidemiologic links: cases confirmed to be part of an outbreak (e.g., “o12 confirmed” – case part of outbreak o12), cases designated as possibly part of an outbreak, and cases designated as sporadic.

All pool-linked cases were caused by *C. hominis* subtypes IeA11G3T3, IaA12R3, or IdA15G1 (Table 3). The childcare outbreak case involved *C. parvum* subtype IIaA19G3R1, while the case linked to a camp outbreak was *C. parvum* IIaA20G3R1. Two swimming pool outbreaks, o4 and o12, were linked to two subtypes (Table 3). Seven swimming pool outbreaks were linked to subtype IeA11G3T3 and five to IaA12R3. Other subtypes (e.g., IfA12G1R5, IaA16R4, IIIjA4T1R1, IIIgA31G4R1, OTUi A12G1, IIaA18G3R1, IIaA16G1R1) were identified in sporadic cases. Based on spatiotemporal overlap and shared subtypes, 52 additional cases not formally linked to outbreaks were inferred to represent undetected outbreak-associated infections (Fig. 3; Table 3). Two cases could potentially be linked to two different outbreaks: o18 or o20 and another either o4 or o23. The case infected with *C*. sp. OTUi was a female between the ages of 21 and 30. The cases infected by *C. fayeri* and *C. occultus* were not notified and found in a female (61–70 years old) and a male (0–10 years old), respectively, both from the same regional area.

### Australia ranks among the top three countries with the largest rise in cryptosporidiosis cases in 2023–2024

**Fig 4:**
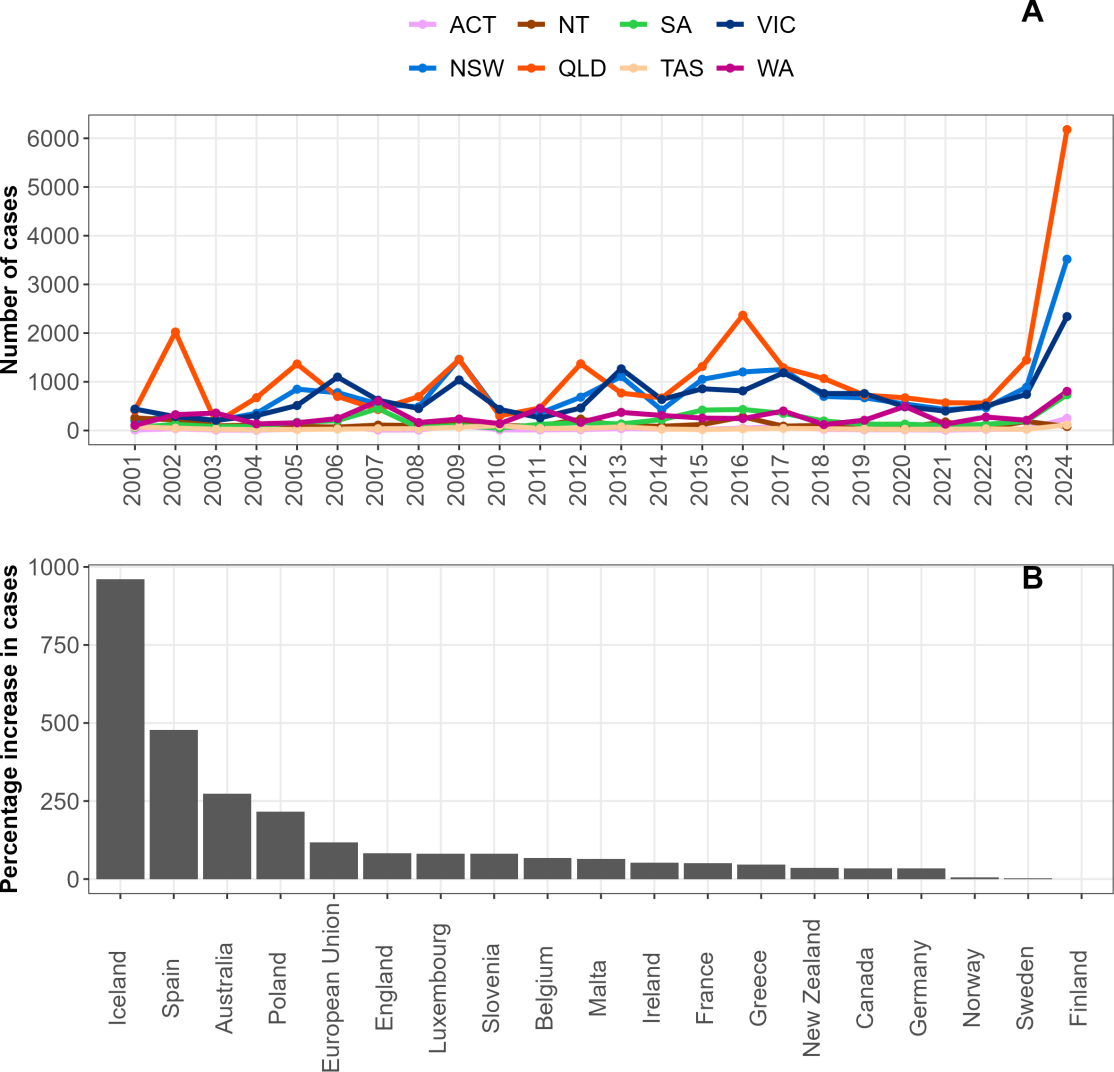
Cryptosporidiosis cases in Australia and abroad. (**A**) Annual number of notified cases in each Australian jurisdiction. (**B**) Percentage of increase in reported cases from 18 countries (and the European Union as a whole) for the 2023/2024 period compared to the previous year. Abbreviations for Australian states and territories: ACT, Australian Capital Territory; NSW, New South Wales; NT, Northern Territory; QLD, Queensland; SA, South Australia; TAS, Tasmania; VIC, Victoria; WA, Western Australia.

Nationally, Australia recorded a 273% increase in cases in 2024 compared to 2023. Except for the Northern Territory (NT), the number of cases in 2024 from all Australian jurisdictions showed a >200% increase (Fig. 4A). The highest percentage of case increase was in the Australian Capital Territory, Tasmania, and Queensland, with 793%, 360%, and 327% respectively. The highest case numbers were in the Eastern states of Queensland, New South Wales, and Victoria. Apart from NT, the number of cases reported in 2024 in all jurisdictions was the highest ever recorded since cryptosporidiosis became notifiable in 2001. Internationally, Australia placed third, behind Iceland and Spain, among countries reporting the sharpest increase in cases in 2023–2024 (Fig. 4B; Table S1). A sharp increase in cryptosporidiosis cases in 2023–2024 was also observed in England, Ireland, Belgium, Poland, France, Luxembourg, Slovenia, Malta, and Czechia. Australia was the only non-European country to record a sharp increase in cryptosporidiosis during this period. There was no geographical pattern within the European countries that reported a sharp increase in cases. Moderate increases were observed in New Zealand, Canada, Portugal, Latvia, Greece, and Germany. The USA was excluded due to unpublished data for 2023 and 2024.

## DISCUSSION

This study presents the first comprehensive molecular investigation of *Cryptosporidium* and *Giardia* infections in humans in the state of Victoria, Australia. Of 2,330 fecal samples analyzed, 225 were test-positive for *Cryptosporidium* and nine for *Giardia*. The screening of 2,102 untested symptomatic cases did not reveal evidence of large cryptic outbreaks. Low detection rates in 2020–2022, observed here and in other countries, likely reflect reduced transmission due to COVID-19 public health restrictions (97, 98). In 2024, despite multiple cryptosporidiosis outbreaks, there was a very low number of cases and no evidence of large cryptic outbreaks related to notified foodborne or waterborne outbreaks, based on the untested cases screened in this study.

### Novel taxa within three *Cryptosporidium* species

Three *Cryptosporidium* subtypes identified in this study are taxonomically uncertain and may represent novel lineages. The *C. meleagridis* subtype IIIA19G1 is genetically distinct and not assigned to a known subfamily. The *C. hominis* IbA8G4 sequence differs markedly from the only other published IbA8G4 record and sits on a long branch in the Ib clade, potentially indicating a divergent subfamily. Subtype A12G1 of *C*. sp. OTUi is novel, with a similar subtype from the traveler returning from Indonesia (2014), but no *gp60* sequence from the bat in the Philippines (2025).

The *Cryptosporidium* sp. “mink genotype” detected in three patients in 2020 is also distinct compared to other *C.* sp. mink genotypes from abroad. The *SSU* and *gp60* sequences from these cases clustered separately from all known animal-derived sequences, supporting the possibility of a regionally divergent Australian lineage. Previous reports in Australians (88, 89) also revealed ambiguous placement in phylogenies. Notably, all three cases here occurred within 48 h during the strict COVID-19 lockdown and were recorded within different households situated in two suburbs of Melbourne, which limited possible exposure routes. The immune status of these individuals is unknown; one of the cases also tested positive for *Staphylococcus aureus*. Overall, the infection source, transmission route, and taxonomic position of this genotype remain unresolved.

### First reports of human infection and subtypes in Australia

This study identified three *Cryptosporidium* subtypes not previously detected in the human host—namely *C. occultus* XXIVa, *C. fayeri* IVfA10G1T1, and *C. meleagridis* IIIjA4T1—previously found only in rats, macropods, and rock-wallabies, respectively (80, 94, 95). The identification of *C. meleagridis* IIIjA4T1 in a human raises questions about potential zoonotic or anthroponotic exchange in captive marsupial settings, as well as the issue of parasite detection in likely passive hosts from zoos and other captive environments. Subtype XXIVa is the first record in a human, with previous records reported in animals (mostly rats and cattle) from Sweden (80).

Additionally, seven subtypes were reported for the first time in Australia, including *C. meleagridis* IIIgA31G4. Six of the 10 *C. hominis* subtypes (IaA12R3, IaA14R3, IaA16R3, IaA16R4, IaA24R1, IfA13G1R4) had not been documented in Australia. The highly common subtype IaA12R3 found in this study, which has been associated with multiple swimming pool outbreaks, had not been previously linked to outbreaks worldwide (99). In contrast, all *C. parvum* subtypes and *Giardia* identified herein had been reported previously.

### Subtype dynamics and cryptic outbreak

*Cryptosporidium hominis* was the dominant species (85%), consistent with prior Australian reports (30, 39) and with observations from South Asia and sub-Saharan Africa (100, 101). This contrasts with Europe and North America, where *C. parvum* is more common (83, 102–104).

The most frequent subtypes were IeA11G3T3 (41%) and IaA12R3 (23%). While both have been reported previously, neither has been linked to large outbreaks (99). Subtype IeA11G3T3 has recently become common in the USA, Norway, Sweden, New Zealand, and India. It is the second most dominant subtype across the American continent and Bangladesh, and the dominant subtype in Australia, Scotland, Israel, Canada, and Zambia (7, 42, 100–102, 105–111). Subtype IfA12G1R5, found in 5% of samples in this study, has caused outbreaks in the USA and Spain (5, 72, 73, 107) and was dominant in Western Australia in 2017 (112). It has also been commonly reported from New Zealand and Europe (4, 105, 113). Although no outbreak linkage was identified here for IfA12G1R5, its frequency suggests a cryptic transmission cluster. The previously dominant IbA10 subtype was absent, which may reflect evolving strain competition or incomplete sample referral. Subtype IdA15G1 has previously been linked to swimming pool outbreaks in the USA (114).

Only *C. parvum* subfamily IIa was detected, consistent with zoonotic exposure. Seven subtypes were recorded, several of which (e.g., IIaA16G1R1, IIaA18G3R1, IIaA20G3R1) have been associated with livestock or wildlife in Australia. The absence of subtype IIaA15G2R1, which predominates in Europe and North America (115), further illustrates regional divergence in subtype distribution.

### Zoonotic and environmental infection risk

Multiple subtypes identified here have previously been reported in a restricted host range in Australia (e.g., *C. occultus* in deer, IVfA10G1T1 and IIaA19G4R1 in kangaroos, IIaA16G1R1 in cattle). Internationally, these same subtypes have been detected in diverse hosts, suggesting broad zoonotic potential (41, 116). Thus, local infections may arise via zoonotic or environmental transmission or overseas acquisition. Ongoing One Health surveillance across humans, animals, and environments is essential to clarify source attribution.

Molecular surveillance should incorporate spatiotemporal and epidemiological data, including travel history. The absence of travel information in this data set limits source inference. The case of *C*. sp. OTUi, previously reported in a traveler from Indonesia and a Southeast Asian bat, highlights the importance of contextualizing findings with travel and wildlife exposure.

### Quantification and transmission dynamics

Oocyst loads estimated by qPCR were consistent with previous reports from Australian patients (117, 118). No species or subtype-specific differences in load were observed, suggesting that all are capable of high shedding and transmission. Higher loads were observed in children, supporting prior studies indicating increased susceptibility and transmission potential in young age groups (119, 120). Oocyst loads did not appear to be a marker of disease severity based on hospitalization data available. However, hospitalization is likely to be under-reported, as not all sporadic cases could be followed up due to limited resources at the time.

### Outbreaks and public health risk

Recurring outbreaks linked to swimming pools are well documented in Australia (18–30). The continued detection of *C. hominis* subtypes in pool-linked outbreaks here highlights persistent issues in infection prevention and water quality control. Risk factors include pool design, filtration efficacy, hygiene compliance, and microbiological monitoring (121, 122).

Subtype overlap across geographically dispersed outbreaks suggests wider or sustained transmission. Notably, IeA11G3T3 and IaA12R3 were involved in 11 outbreaks and identified in many other sporadic cases from adjacent LGAs during the same period, suggesting potential under-detection of outbreak scale. The detection of two different subtypes within outbreaks suggests either that the cases belong to two different outbreaks or that a mixed population served as the outbreak’s initial infection source. The two outbreaks linked to *C. parvum* suggest a primary zoonotic infection source and possibly secondary anthroponotic transmission between children. Subtype IIaA19G3R1 and IIaA20G3R1 have previously been recorded in Victoria from deer, alpaca, and cattle hosts (62, 123, 124). Many *C. parvum* subtypes (e.g., IIaA18G3R1, IIaA19G4R1) occurred in multiple sporadic cases and may represent cryptic zoonotic outbreaks. Strengthening the One Health approach would improve identification of such linkages.

This study presents the first Australian molecular and epidemiological data from the 2023–2024 period, which saw multiple outbreaks across Australia and the highest number of cases recorded in the last 24 years. Additional data from that period from other jurisdictions would allow further assessment of the scale of *Cryptosporidium* transmission and identify common sources of infection. Previous reviews have found a greater number of *Cryptosporidium* outbreaks from North America and New Zealand (125, 126) compared to other regions, suggesting a different disease dynamic during the 2023–2024 period; however, the lack of data from the USA limits interpretation for North America. The role of swimming pools in other Australian jurisdictions has yet to be quantified and compared to the Victorian data. The sharp increase in cases observed in several European countries suggests that common risk factors specifically increased during this period. The European studies published indicated outbreaks also linked to swimming pools and international travel (4–8). Splash pads or parks were not identified as a source of outbreak in this period in Australia or abroad. The rise of cases globally during the 2023-2024 summer could be due to this period being one of the warmest on record (127–129). Cryptosporidiosis infections have been shown to increase in warmer months (16, 103, 130), suggesting an increasing risk from this disease in the future. The association between increased temperature and increased cryptosporidiosis cases linked to swimming pools indicates a need for public health authorities to increase prevention and surveillance of cryptosporidiosis in this setting globally. The association with international travel and the worldwide increase in cases suggests that outbreaks could occur on an increasingly international scale. Worldwide transmission of *C. parvum* subtype has been documented previously (115).

### Genotyping methods and future directions

The *gp60* gene is the most widely used subtyping marker, but it has limited discriminatory power for outbreak resolution (107, 131, 132). This study supports the use of qPCR to rapidly screen clinical samples, as previously recommended (133). The low success rate of *Giardia* sample identification and quantification in this study could be due to sample degradation, targeted loci sensitivity issues, and the presence of amplification inhibition. Other qPCR target loci are available and could be employed to evaluate the cause of the quantification failure observed here (134, 135).

Higher-resolution tools, including MLVA (136), multilocus sequence typing (137), RNA-bait (138), and whole-genome sequencing (115, 131, 139), are increasingly being used. However, few are routinely applied to the full taxonomic range of *Cryptosporidium* infecting humans. Whole-genome sequencing offers the highest resolution but has not yet been adopted for routine typing. Given its status as a nationally reportable disease in Australia, PCR-based subtyping tools, employing *SSU* and *gp60* as markers, are already available, practical, and should be systematically applied to notified cases of cryptosporidiosis.

### Conclusion

This study identified seven *Cryptosporidium* species and 24 subtypes, as well as four *Giardia* assemblages from clinical fecal samples in Victoria over five years. The observed diversity and subtype distribution suggest multiple transmission pathways, including undetected outbreaks and zoonotic reservoirs, operating concurrently. High sequence identity with animal and environmental isolates supports the need for integrated One Health surveillance. The subtyping of clinical samples, combined with linked metadata, provides a solid foundation for improving the control and prevention of cryptosporidiosis and giardiasis in Australia.

## Data Availability

Sequences are deposited in NCBI under accession PV981441–PV981665 (*SSU*), PX092398–PX092617 and PX048014 (*gp60*), and PX048005–PX048013 (*tpi*).

## SUPPLEMENTAL MATERIAL

Supplemental figures jcm.01558-25-s0001.docx Figures S1 to S5.

Supplemental material jcm.01558-25-s0002.docx Supplemental methods.

Table S1 jcm.01558-25-s0003.xlsx Worldwide reported cryptosporidiosis cases from 2019 to 2024.

ASM does not own the copyrights to Supplemental Material that may be linked to, or accessed through, an article. The authors have granted ASM a non-exclusive, world-wide license to publish the Supplemental Material files. Please contact the corresponding author directly for reuse.
