# Supplementary material for: Large-scale molecular epidemiological survey of Giardia and Cryptosporidium in Victoria, Australia (2020–2024), reveals novel subtypes and outbreak-associated lineages
Source: J Clin Microbiol. 2026 Mar 27;64(5):e01558-25. doi: 10.1128/jcm.01558-25 (PMC13170286; doi:10.1128/jcm.01558-25)
Supplement: Supplemental figures — Figures S1 to S5. [file jcm.01558-25-s0001.docx]

**Large-scale molecular epidemiological survey of *Giardia* and *Cryptosporidium* in Victoria, Australia (2020-2024) reveals novel subtypes and outbreak-associated lineages.**

Babineau et al.

**Supplementary Figures**


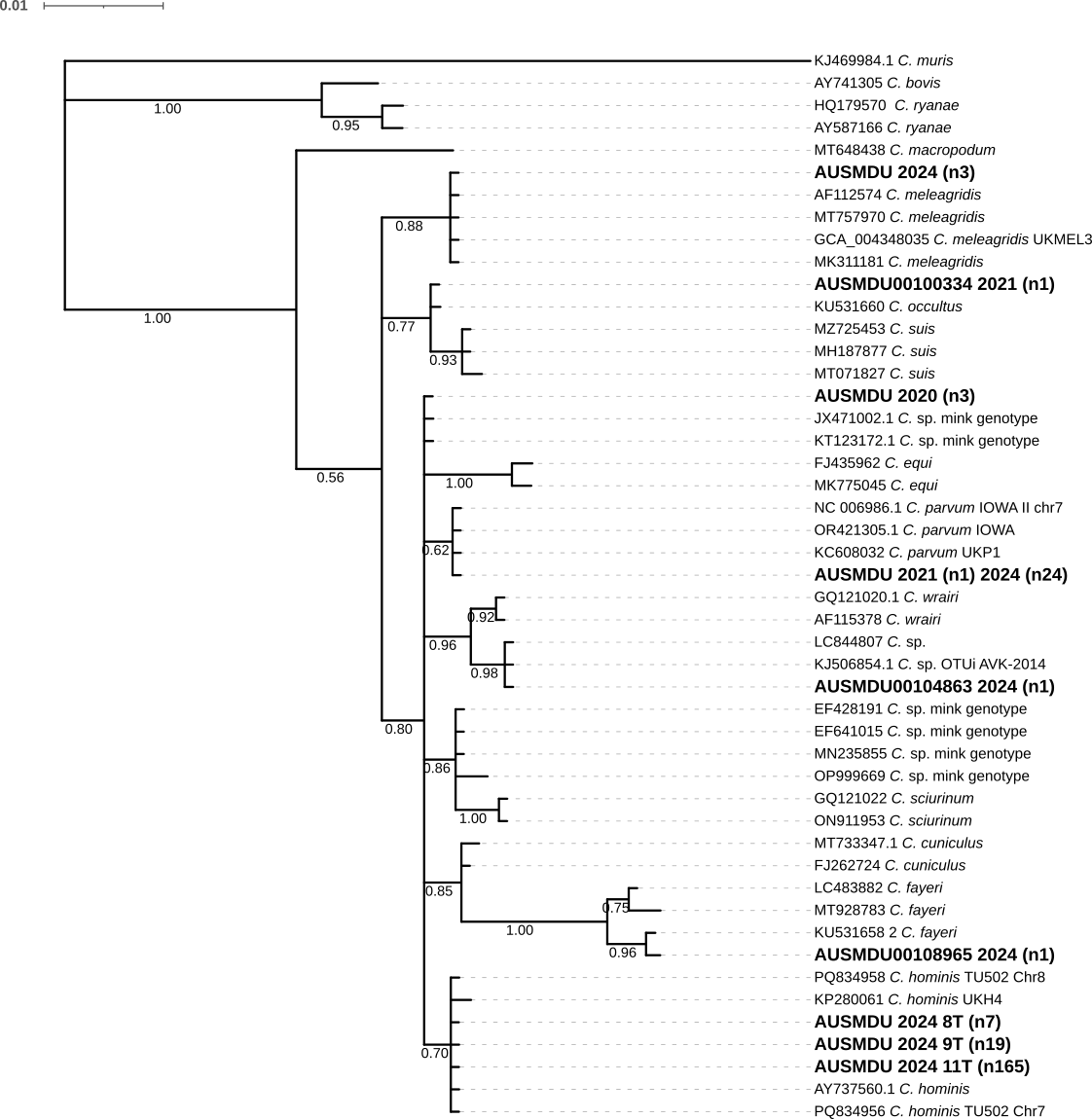


Figure S1. Phylogenetic tree reconstructed from the bayesian topology using GTR +G+I model based on the dereplicated alignment of the small subunit ribosomal RNA gene (*SSU*) with 10M generations for *Cryptosporidium*. Sequences generated in this study are shown in bold with the number of samples identified within parenthesis (n). Posterior probabilities are shown below the branch. *Cryptosporidium muris* used as outgroup.


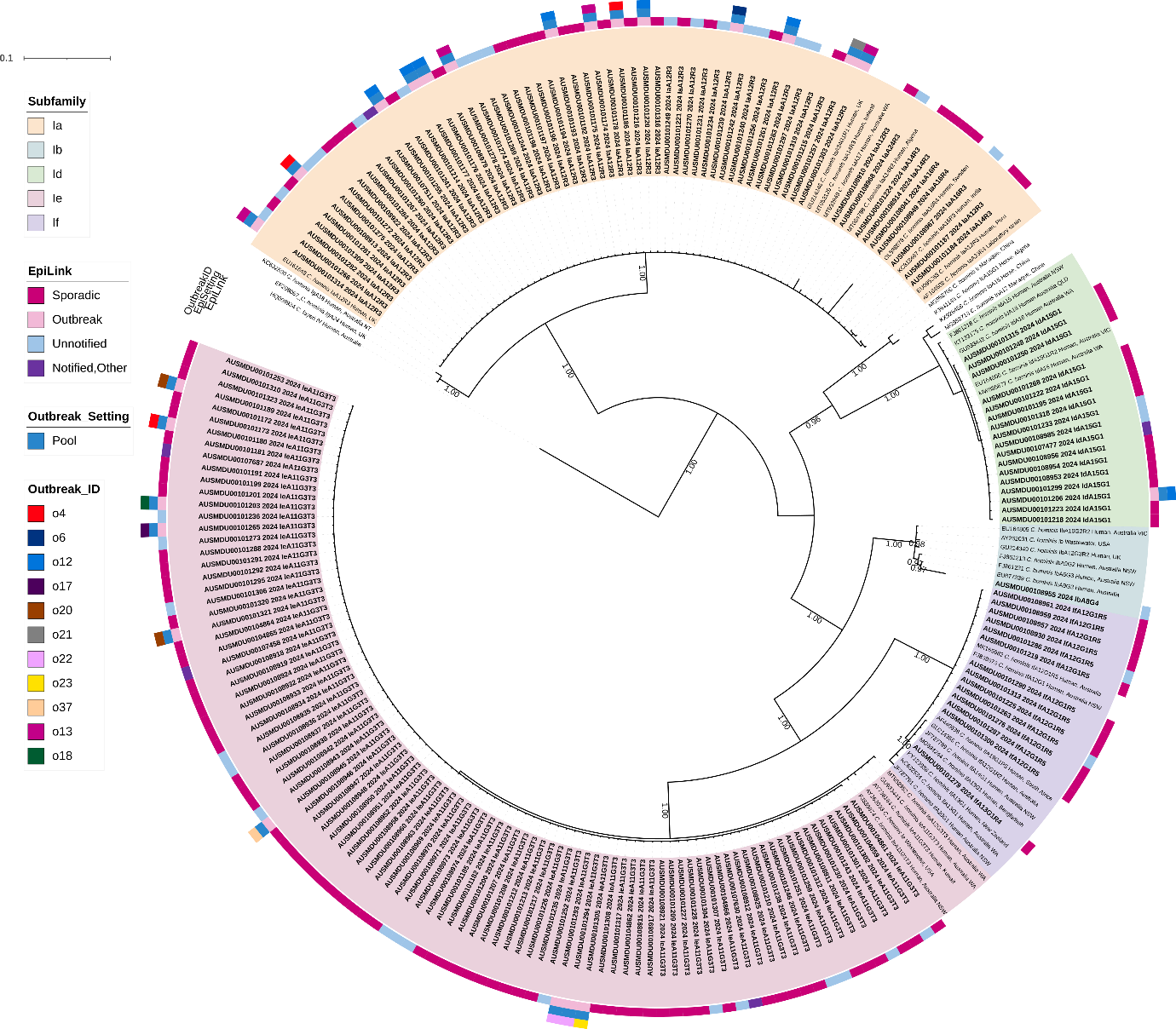


Figure S2. Phylogenetic tree reconstructed from the bayesian topology using GTR +G+I model based on 60kD glycoprotein gene (*gp60*) with 50M generations for *C. hominis* subtypes. Sequences generated in this study are shown in bold. Posterior probability equal to or above 0.90 are shown below the branch. *Cryptosporidium fayeri* used as outgroup. Taxa labels highlighted by *gp60* subfamily identity. Additional data shown as coloured strips; epidemiological link (Epi Link), outbreak setting, outbreak identification number (Outbreak ID). Epi Link: cases classified as sporadic, outbreak related, not notified to Victorian Department of Health as of 18/06/2025.


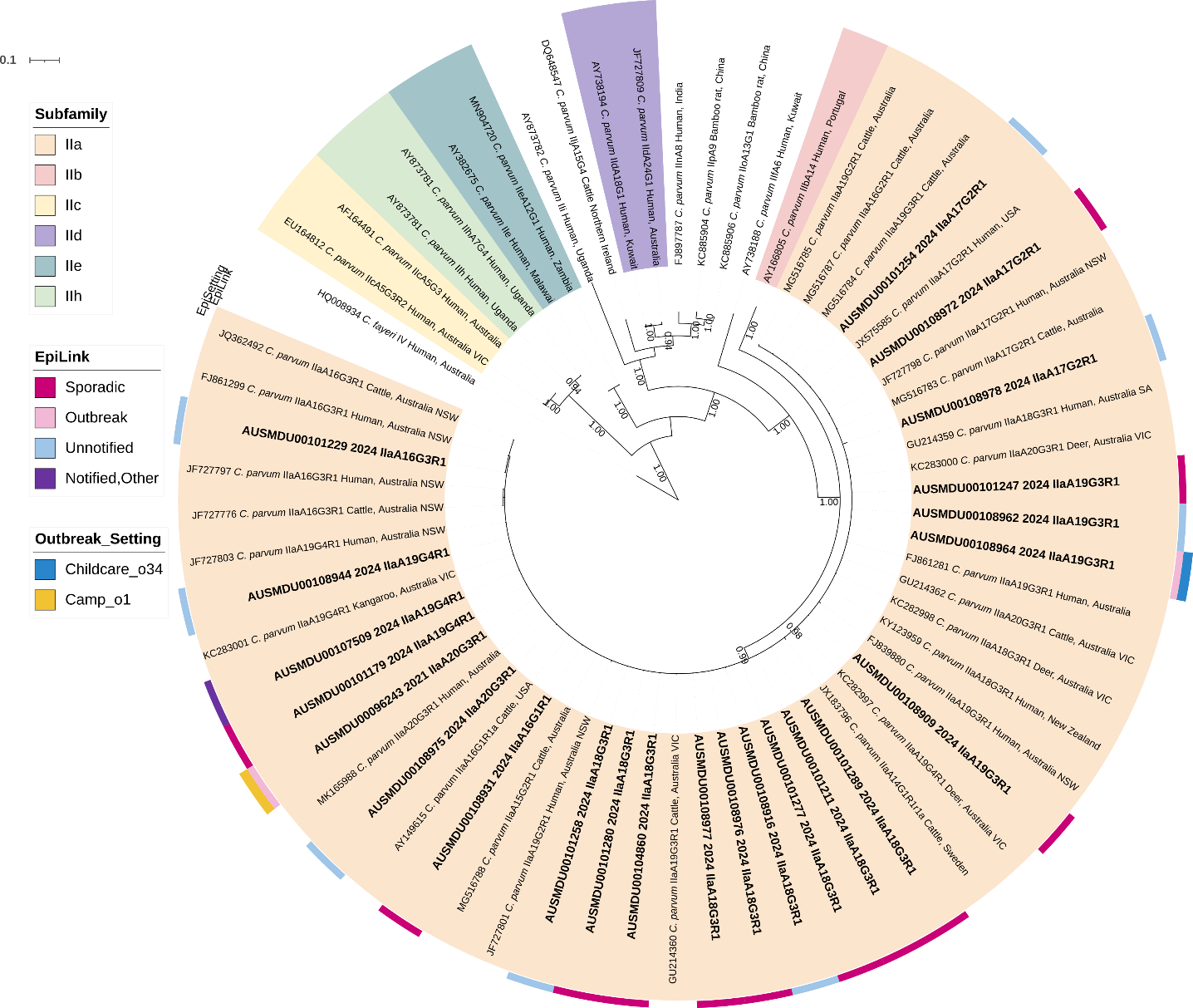


S3. Phylogenetic tree reconstructed from the bayesian topology using GTR +G+I model based on 60kD glycoprotein gene (*gp*60) with 50M generations for *C. parvum* genotypes. Sequences generated in this study are shown in bold. Posterior probability equal to or above 0.90 are shown below the branch. *Cryptosporidium fayeri* used as outgroup. Additional sample data shown as coloured strips; epidemiological link (Epi Link), outbreak setting and identification number (Outbreak Setting).


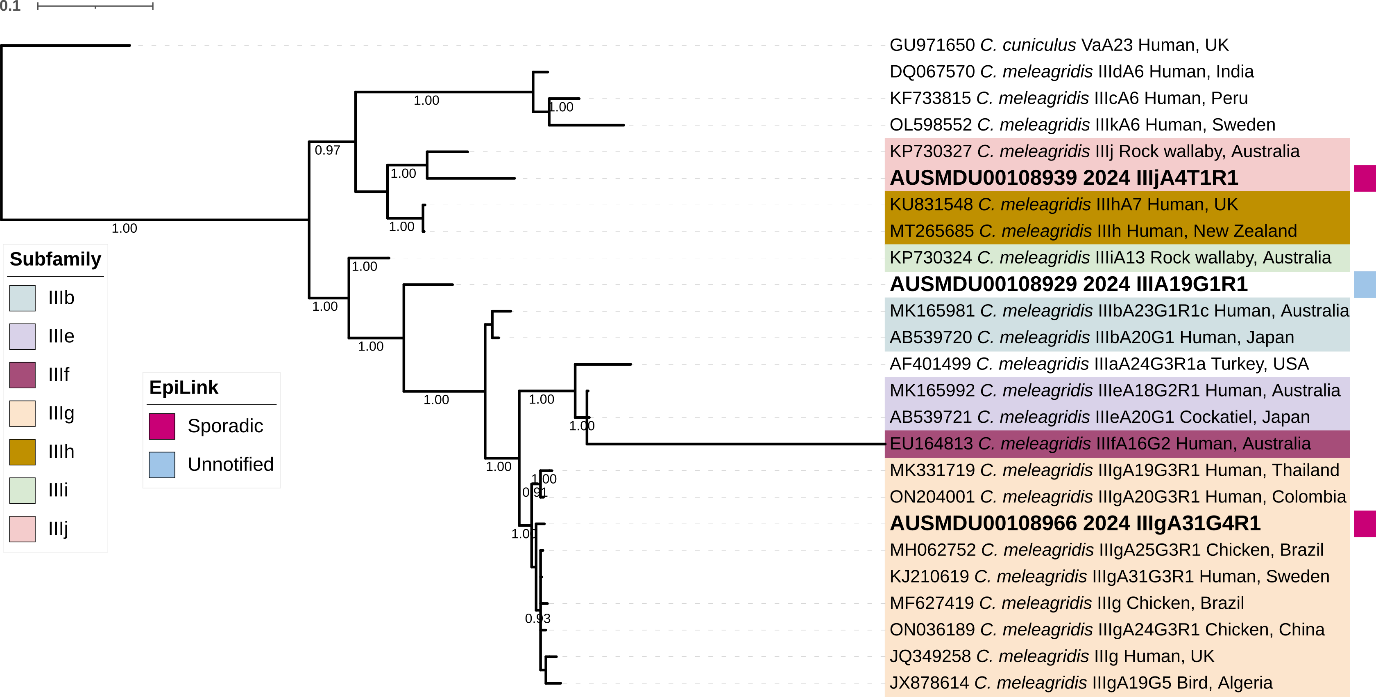


S4. Phylogenetic tree reconstructed from the bayesian topology using GTR +G+I model based on 60kD glycoprotein gene (*gp*60) with 10M generations for *C. meleagridis* sequences. Sequences generated in this study are shown in bold. Posterior probability equal to or above 0.90 are shown below the branch. *Cryptosporidium cuniculus* used as outgroup. Additional sample data shown as coloured strips; epidemiological link (Epi Link).


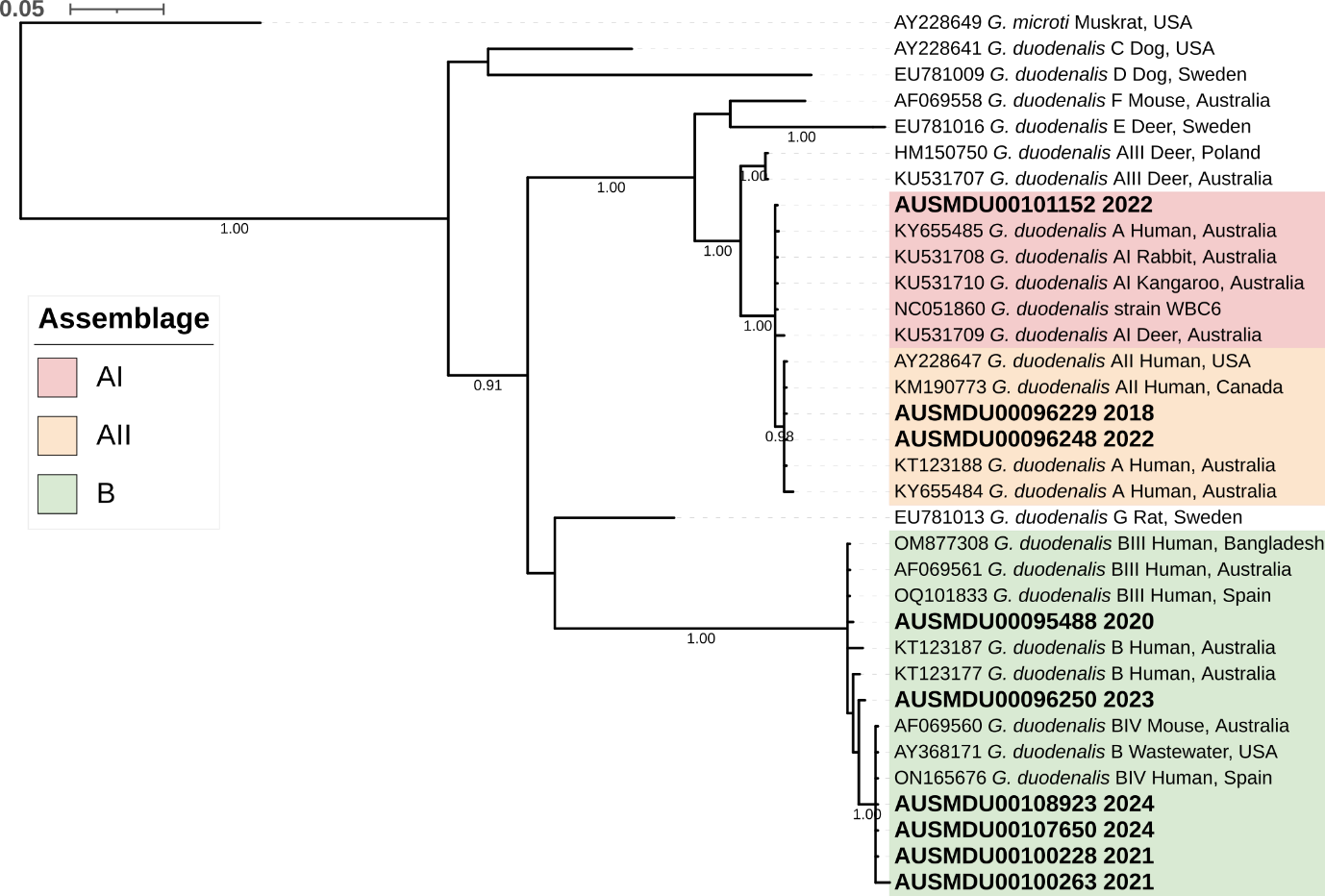


S5. Phylogenetic tree reconstructed from bayesian topology using K2 +G+I model based on triose phosphate isomerase (*tpi*) loci with 10M generations for *Giardia* species. Sequences generated in this study are shown in bold. Posterior probability equal to or above 0.90 are shown below the branch. *Giardia microti* used as outgroup.
