# Supplementary material for: Large-scale molecular epidemiological survey of Giardia and Cryptosporidium in Victoria, Australia (2020–2024), reveals novel subtypes and outbreak-associated lineages
Source: J Clin Microbiol. 2026 Mar 27;64(5):e01558-25. doi: 10.1128/jcm.01558-25 (PMC13170286; doi:10.1128/jcm.01558-25)
Supplement: Supplemental material — Supplemental methods. [file jcm.01558-25-s0002.docx]

**Large-scale molecular epidemiological survey of *Giardia* and *Cryptosporidium* in Victoria, Australia (2020-2024) reveals novel subtypes and outbreak-associated lineages.**

Babineau et al.

**Supplementary Methods**

**Multiplex qPCR**

A multiplex qPCR assay was developed and tested to be used as a detection and quantification method for *Cryptosporidium* oocysts and *Giardia* cysts in faecal samples. Within this multiplex (*gdh*, *SSU*) assay, *Cryptosporidium* oocysts were quantified targeting *SSU* using qPCR primers and probe described previously (Morgan et al. 1997; King et al. 2005; Yang, Paparini, et al. 2014). The probe was modified to use the HEX fluorophore. To detect and quantify *Giardia*, the glutathione dehydrogenase (*gdh*) gene was targeted using the primers and probe from Yang et al (Yang, Jacobson, et al. 2014) with the probe modified to use the FAM-fluorophore. The primers and probe mix to detect the RT-qPCR Extraction control red were used (Meridian, USA). Each 20 µl qPCR reaction contained 10 µl of the 2x KAPA Fast Probe master mix (KAPA), 0.8 µl of BioRED mastermix, 400 nM of each *gdh* primers, 250 nM of *gdh* probe, 500 nM of each primer and probe targeting *SSU*, topped to 20 µl with deionised H_2_0, and 2 µl of DNA. Thermocycler (BioRAD CFX-96) conditions were: initial denaturation at 95 °C for 3 min, followed by 45 cycles at 95 °C for 10 sec and 30 sec at 60 °C. Detection was set to FAM, HEX and Cy5 with a baseline threshold of 100 RFU for each channel.

Purified *Cryptosporidium* oocysts and *Giardia* trophozoites were obtained to generate enumerated serial dilutions to be used as quantification reference for the qPCR. *Cryptosporidium* *parvum* oocyst were obtained (Bunchgrass Farm, Iowa, USA). DNA was extracted using approx. 40 million oocysts. Seven freeze thaw cycles (liquid nitrogen for 2 min, 3 min at 70 °C) were performed followed by homogenisation by passing through 25G syringe ten times. Proteinase K (Promega, USA) was added to a final concentration of 400 µg/mL and incubated overnight at 55°C followed by 1h incubation at 37 °C with 50,000 unit/mL of RNAse If (New England Biolabs, Australia). DNA was purified using two Phenol:Chloroform:Isoamyl 24:24:1 (Thermo Fisher Scientific, USA) purification steps. DNA was precipitated in isopropanol for 1h and pellet washed in 80% ethanol. Approximately 20 million trophozoites of *Giardia* *duodenalis* were extracted using the method described above. A 10-fold serial dilution was prepared for each parasite DNA sample for a total of nine dilutions (1:1 until 1:100M). Based on the DNA concentration from the 1:1 DNA sample and the size of the parasite genomes (9.1 MBp was used for *Cryptosporidium* while 12 MBp was used for *Giardia*), the number of DNA copy number per microliter was calculated using Avogadro’s number. Using a ploidy level of 4, the number of oo/cysts was calculated for each dilution in the series (Ruecker et al. 2011; Nolan et al. 2015).

**Multiplex qPCR validation**

The multiplex assay was validated by comparing the qPCR efficiency (E) and adjusted R^2^ of each probe in the multiplex assay versus singlex using the standard curve dilution series. Both probes showed very similar efficiency in multiplex versus singlex; *gdh* assay: E 106% vs 100%, R^2^ 0.993 vs 0.991 respectively, *SSU* assay: E 92% vs 94%, R^2^ 0.999 vs 0.998 respectively. Across all samples tested, the mean Ct for the RT-qPCR Extraction control red was 28.01 with 17% of samples showing a Ct above 35 or below 25.

A range of species were tested to assess assay specificity. The four most common *G. duodenalis* sub-assemblages were used to ensure detection for *gdh* assay and exclusion for *SSU* assay. Eleven species of *Cryptosporidium*, representing a wide taxonomic range, were used to ensure detection for *SSU* assay and exclusion for *gdh* assay; *C. muris* (1), *C. cuniculus* (2), *C. meleagridis* (2), *C. parvum* (2), *C. ryanae* (1), *C. fayeri* (1), *C. hominis* (2), *C. macropodum* (2), *C. occultus* (1), and *C. ubiquitum* (2). Three bacterial genera commonly found in human faecal samples were also used to ensure exclusion for both assays; *Collinsella, Phocaeicola* and *Bifidobacterium*. The *gdh* assay detected *G. duodenalis* AI and AII (3/4 samples detected) and detected *G. duodenalis* assemblage B (5/5 samples detected). The *gdh* assay did not detect the ten *Cryptosporidium* species and did not detect the three bacterial faecal species. The *SSU* assay detected all species of *Cryptosporidium* and did not detect any of the *Giardia* samples, and did not detect the bacterial faecal species.

The limit of detection (LoD) of the multiplex assay was determined based on screening 10-fold serially diluted DNA from the respective enumerated sample using eight replicates per dilution. The LoD was determined to be the amount of DNA that can be reliably detected (100% of the eight replicates). The corresponding Cts were used as the threshold to include samples for further oo/cyst quantification. The LoD was determined to be 0.00315 pg/µl of DNA for the *SSU* probe (Ct of 37.9) and 0.0271 pg/µl of DNA for the *gdh* probe (Ct of 37.0).

**Oo/cyst load quantification**

The number of *Cryptosporidium* oocysts and *Giardia* cysts were estimated using the multiplex qPCR validated above in combination with the enumerated dilution series standards generated as described above. PCR-positive samples were screened along with five 10-fold dilution of the enumerated series of the *Giardia* or *Cryptosporidium*. Samples showing a Ct value above 37 were removed. All samples were screened in triplicate. Species and subtypes were compared using ANOVA based on oo/cyst number when there was a minimum of three or more quantified samples for each taxon. To confirm the qPCR quantification, three fresh *Cryptosporidium*-positive faecal samples were purified using immunomagnetic separation (CryptoBeads; BioPoint Ltd Pty, Sydney, Australia) followed by enumeration in a hemocytometer under a light microscope.

**Comparison of cryptosporidiosis cases nationally and internationally**

The number of notified cryptosporidiosis cases in Australia and for all states and territories were retrieved from the Australian National Notifiable Disease Surveillance System (NNDSS) data visualisation tool, [www.nindss.health.gov.au](http://www.nindss.health.gov.au) [accessed on August 7^th^ 2025]. Cases from England were retrieved from the UK Health and Security Agency reports (UK Health Security Agency 2025a; 2025b). Cases from the United States of America were retrieved from U.S. Centers for Disease Control and Prevention (CDC) WONDER online database [www.wonder.cdc.gov](http://www.wonder.cdc.gov) , the National Notifiable Disease Surveillance System (NNDSS) annual data tables option was selected, from which the interactive NNDSS annual summary data query and the CDC Stacks were accessed [accessed on August 7^th^ 2025]. The total number of cryptosporidiosis cases was selected. Cases from the European Union were retrieve from the European Centre for Disease Prevention and Control (ECDC) Surveillance Atlas of Infectious Disease online tool [www.atlas.ecdc.europa.eu](http://www.atlas.ecdc.europa.eu) [accessed on August 7^th^ 2025]. The number of EU (without the UK for 2019) confirmed reported cases were retrieved for each year. Within the EU, country-level data was also obtained from Bulgaria, Belgium, Croatia, Cyprus, Czechia, Estonia, Finland, France, Greece, Germany, Hungary, Ireland, Italy, Iceland, Latvia, Lithuania, Luxembourg, Malta, Norway, Netherlands, Poland, Portugal, Romania, Spain, Slovenia, Slovakia, and Sweden. Some The number of cases from France before 2023 were obtained from the French National Network on Surveillance of Human Cryptosporidiosis (D. Costa, F. Dalle, S. Basmaciyan and L. Favennec, pers. comm). Cases from New Zealand were retrieved from New Zealand Institute for Public Health and Forensic Science (PHF Science) Notifiable Disease Dashboard [www.phfscience.nz/digital-library/notifiable-disease-dashboard/](http://www.phfscience.nz/digital-library/notifiable-disease-dashboard/) [accessed on August 7^th^ 2025]. Cases from Canada were retrieved from the Public Health Agency of Canada Notifiable Diseases Online [www.diseases.canada.ca/ndis/](http://www.diseases.canada.ca/ndis/) [accessed on August 7^th^ 2025].

**References**

King, Brendon J., Alexandra R. Keegan, Paul T. Monis, and Christopher P. Saint. 2005. “Environmental Temperature Controls *Cryptosporidium* Oocyst Metabolic Rate and Associated Retention of Infectivity.” *Applied and Environmental Microbiology* 71 (7): 3848–57. https://doi.org/10.1128/AEM.71.7.3848-3857.2005.

Morgan, Una M., Clare C. Constantine, David A. Forbes, and R. C. Andrew Thompson. 1997. “Differentiation between Human and Animal Isolates of Cryptosporidium Parvum Using rDNA Sequencing and Direct PCR Analysis.” *The Journal of Parasitology* 83 (5): 825. https://doi.org/10.2307/3284275.

Nolan, Matthew J., Fiona M. Tomley, Pete Kaiser, and Damer P. Blake. 2015. “Quantitative Real-Time PCR (qPCR) for Eimeria Tenella Replication — Implications for Experimental Refinement and Animal Welfare.” *Parasitology International* 64 (5): 464–70. https://doi.org/10.1016/j.parint.2015.06.010.

Ruecker, Norma J., Rebecca M. Hoffman, Rachel M. Chalmers, and Norman F. Neumann. 2011. “Detection and Resolution of Cryptosporidium Species and Species Mixtures by Genus-Specific Nested PCR-Restriction Fragment Length Polymorphism Analysis, Direct Sequencing, and Cloning.” *Applied and Environmental Microbiology* 77 (12): 3998–4007. https://doi.org/10.1128/AEM.02706-10.

UK Health Security Agency. 2025a. *Cryptosporidium Data 2014 to 2023*. Research and analysis. Gastrointestinal Infections, Food Safety and One Health Division. https://www.gov.uk/government/publications/cryptosporidium-national-laboratory-data/cryptosporidium-data-2014-to-2023.

UK Health Security Agency. 2025b. *Cryptosporidium Data 2015 to 2024*. Research and analysis. Gastrointestinal Infections, Food Safety and One Health Division. https://www.gov.uk/government/publications/cryptosporidium-national-laboratory-data/cryptosporidium-data-2015-to-2024.

Yang, Rongchang, Caroline Jacobson, Graham Gardner, Ian Carmichael, Angus J.D. Campbell, and Una Ryan. 2014. “Development of a Quantitative PCR (qPCR) for Giardia and Analysis of the Prevalence, Cyst Shedding and Genotypes of Giardia Present in Sheep across Four States in Australia.” *Experimental Parasitology* 137 (February): 46–52. https://doi.org/10.1016/j.exppara.2013.12.004.

Yang, Rongchang, Andrea Paparini, Paul Monis, and Una Ryan. 2014. “Comparison of Next-Generation Droplet Digital PCR (ddPCR) with Quantitative PCR (qPCR) for Enumeration of Cryptosporidium Oocysts in Faecal Samples.” *International Journal for Parasitology* 44 (14): 1105–13. https://doi.org/10.1016/j.ijpara.2014.08.004.
